# Role of Metalloproteinases in Diabetes-associated Mild Cognitive Impairment

**DOI:** 10.2174/1570159X22666240517090855

**Published:** 2024-07-03

**Authors:** Vitoria Mattos Pereira, Suyasha Pradhanang, Jonathan F. Prather, Sreejayan Nair

**Affiliations:** 1 School of Pharmacy, College of Health Sciences, Biomedical Sciences, Interdisciplinary Graduate Program, University of Wyoming, Laramie, WY 82071, USA;; 2 Department of Zoology and Physiology, Program in Neuroscience, University of Wyoming, Laramie, WY 82071, USA

**Keywords:** Mild cognitive impairment, T2DM, dementia, MMPs, diabetes, inflammation

## Abstract

Diabetes has been linked to an increased risk of mild cognitive impairment (MCI), a condition characterized by a subtle cognitive decline that may precede the development of dementia. The underlying mechanisms connecting diabetes and MCI involve complex interactions between metabolic dysregulation, inflammation, and neurodegeneration. A critical mechanism implicated in diabetes and MCI is the activation of inflammatory pathways. Chronic low-grade inflammation, as observed in diabetes, can lead to the production of pro-inflammatory cytokines such as tumor necrosis factor-alpha (TNF-α), interleukin-6 (IL-6), interleukin-1 beta (IL-1β), and interferon-gamma (IFNγ), each of which can exacerbate neuroinflammation and contribute to cognitive decline. A crucial enzyme involved in regulating inflammation is ADAM17, a disintegrin, and metalloproteinase, which can cleave and release TNF-α from its membrane-bound precursor and cause it to become activated. These processes, in turn, activate additional inflammation-related pathways, such as AKT, NF-κB, NLP3, MAPK, and JAK-STAT pathways. Recent research has provided novel insights into the role of ADAM17 in diabetes and neurodegenerative diseases. ADAM17 is upregulated in both diabetes and Alzheimer's disease, suggesting a shared mechanism and implicating inflammation as a possible contributor to much broader forms of pathology and pointing to a possible link between inflammation and the emergence of MCI. This review provides an overview of the different roles of ADAM17 in diabetes-associated mild cognitive impairment diseases. It identifies mechanistic connections through which ADAM17 and associated pathways may influence the emergence of mild cognitive impairment.

## 1 INTRODUCTION

Diabetes is a chronic, metabolic disease characterized by elevated levels of blood glucose (hyperglycemia), which eventually results in alterations in insulin signaling, leading ultimately to insulin resistance and chronic inflammation. The most common is type 2 diabetes mellitus (T2DM), usually in adults, which occurs when the body becomes resistant to insulin or doesn't make enough insulin due to lifestyle imbalance. According to the World Health Organization (WHO), 422 million people worldwide have diabetes, a number likely to more than double in the next 20 years [1], with an estimated total economic cost of $327 billion [2].

Individuals with T2DM are at an increased risk of mild cognitive impairment (MCI) and dementia [3]. The risk of dementia is increased by 50-100% in people with T2DM relative to people without diabetes [4]. T2DM is associated with mild-to-moderate cognitive deficits, primarily in memory, psychomotor speed, and executive function. Changes in cognitive function compared to non-diabetic controls can be seen early during T2DM [5]. Besides the substantial direct burden that diabetes imposes on society, dementia affects 47 million people worldwide. Every year, there are 9.9 million new cases. The global total of affected people is expected to increase to 75.6 million in 2030 and 135.5 million in 2050 [6].

Persistent hyperglycemia provokes a cascade of physiological alterations throughout the body, including oxidative stress and vascular damage, creating vicious pathological cycling. Elevated blood glucose in uncontrolled diabetes has been attributed to facilitate a low-grade systemic inflammation by causing the elevation of proinflammatory cytokines such as interleukin-6 and C reactive proteins [7-9]. Such low-grade inflammation has been attributed to several long-term complications of diabetes mellitus, including diabetic neuropathy [10, 11], nephropathy [12, 13], and retinopathy [14]. More importantly, cardiovascular disease (both microvascular and macrovascular), for which diabetes is the leading risk factor, has been attributed to the low-grade inflammation caused by chronic uncontrolled diabetes [15, 16]. A recent prospective cohort study of diabetic subjects demonstrated that low-grade inflammation (elevated C-reactive proteins) is an independent risk factor for vascular and all-cause mortality [17]. Additionally, post-hoc analysis of the Modified Release Controlled Evaluation (ADVANCE) population study, the proinflammatory cytokine interleukin-6 was found to be an independent predictor of macrovascular events and mortality [18].

Within the brain, the general low-grade inflammation, insulin resistance, and hyperglycemia compromise neuronal support and exacerbate neuroinflammation [19-21], which collectively impairs cognitive function and ultimately contributes to the development of MCI in diabetic individuals. Although numerous epidemiological and preclinical studies have indicated a strong link between T2DM and cognitive impairment [22, 23], the mechanism of cognitive dysfunction in T2DM remains unclear. It is speculated that the pathological characteristics of diabetes, such as hyperglycemia, insulin resistance, and chronic inflammation, may be associated with structural and pathophysiological changes in the brain leading to cognitive dysfunction [24]. At the molecular level, long-term effects of diabetes have been shown to increase oxidative stress-induced cell death [25, 26]. Furthermore, as insulin receptors are widely expressed in the nervous system [27], impaired insulin signaling in the context of T2DM may also contribute to the development of neurodegeneration [24]. The brain is especially susceptible to oxidative stress due to its high metabolic activity, abundant lipid content and lack of antioxidant enzymes. In addition to the local contribution of hyperglycemia-induced oxidative stress to neuronal inflammation, chronic low-grade systemic inflammation due to uncontrolled hyperglycemia can be a major player in neuronal dysfunction [28]. The circulating proinflammatory cytokines can increase the permeability of the blood-brain barrier, in addition to initiating neuro-inflammation [29]. The cytokines also upregulate the actions of nuclear-transcription factors, causing the transcription, translation, and synthesis of additional pro-inflammatory molecules, feeding into the vicious cycle. Franceschi and coworkers have used the term “inflamm-aging” to describe systemic low-grade inflammation in the context of metabolic disease-associated neuronal aging [30].

Disintegrin and metalloprotease 17 (ADAM17) have recently gained attention due to their pivotal role in a variety of inflammatory conditions [31]. ADAM17 is a shreddase that cleaves and activates a number of cytokines, including tumor necrosis factor-alpha (TNF-α). TNF-alpha directly promotes an inflammatory state and disrupts insulin signaling pathways [32]. In addition to cytokines, ADAM17 is involved in the shredding/processing of chemokines, adhesion molecules, and growth factors [33]. Clinical studies have demonstrated that ADAM17 is overexpressed in biopsies of subjects with chronic inflammatory diseases such as rheumatoid arthritis [34], psoriasis [35], and Crohn’s disease [36]. The treatment of mesangial cells with high glucose results in an elevation of ADAM17 gene expression [37], suggesting a role for ADAM17 in diabetic conditions [38]. ADAM17 has been shown to cleave the ectodomain of the insulin receptor, which can result in insulin resistance [39]. Locally, ADAM17 activates microglia and plays a key role in neuroinflammation [40].

Given the pivotal role of ADAM17 in chronic inflammation, ADAM17 appears to be an attractive treatment target to delay or prevent low-grade chronic neuroinflammation and is associated with the pathophysiology of a number of neurodegenerative diseases. This article provides an overview of the function and regulation of ADAM17 and current knowledge about its role in diabetes and neurodegenerative diseases. In addition, this article examines the involvement of ADAM17 in the molecular pathways of diabetes-associated MCI, highlighting the potential for targeting ADAM17 as a strategic intervention in this condition.

## 2 ADAM17

### 2.1 Structure

ADAM17 is a protease that is part of the ADAM family, which consists of membrane-tethered disintegrin and metalloproteases. These proteases play a significant role in ectodomain shedding, a process involving the cleavage of cell membrane proteins. Among the 30 known ADAMs in mammals, only half possess the metalloproteinase domain and proteolytic potential [41]. ADAM17 shares a highly conserved catalytic domain with other members of the metzincin superfamily, which includes matrix metalloproteases (MMPs) and disintegrin metalloproteinases with thrombospondin domains (ADAMTSs) [42, 43]. The structure of ADAM17 consists of an N-terminal pro-domain, a catalytic domain, a disintegrin domain, a membrane-proximal domain (MPD), and a short stalk domain called CANDIS. It also has a transmembrane domain and an intracellular cytoplasmic domain. The catalytic domain uses a zinc ion (Zn^2+^) for its function, which is coordinated by three histidines in a conserved binding motif [44].

### 2.2 Regulators

Regulation of ADAM17 occurs at multiple levels, including maturation, activity, selectivity, and degradation. A comprehensive understanding of these regulatory mechanisms is essential for elucidating the roles of ADAM17 in different biological contexts and developing potential therapeutic strategies. While this text briefly highlights the regulatory dimension of ADAM17, several previous studies provide a detailed analysis of the regulation of ADAM17 [45-47]. Regarding ADAM17 maturation, this protease is synthesized as an inactive zymogen in the endoplasmic reticulum, which undergoes proteolytic processing in the Golgi apparatus to become an active enzyme. This process involves the removal of the pro-domain by furin-like convertases, which allows the catalytic domain to adopt an active conformation [48]. Additionally, chaperone proteins such as inactive rhomboid protein 1 (iRhom1) and iRhom2 have been shown to facilitate ADAM17 maturation and transport from endoplasmic reticulum to the cell surface [49, 50]. The activity of ADAM17 can be regulated by various factors, including post-translational modifications, protein-protein interactions, and changes in the cellular environment. For instance, phosphorylation of the cytoplasmic domain by different kinases, such as ERK, p38 MAPK [51], and PKC [52], can modulate ADAM17 activity. Moreover, the interaction of ADAM17 with other proteins, such as TIMP3, can inhibit its proteolytic activity [53, 54]. Additionally, changes in the cellular environment, such as oxidative stress, can also affect ADAM17 activity [49]. ADAM17 recognizes and cleaves a wide range of substrates, including cytokines, growth factors, and cell adhesion molecules. Its substrate selectivity is determined by the specific recognition of certain amino acid sequences in the target proteins and the spatial and temporal distribution of both the enzyme and its substrates. Furthermore, substrate availability and competition between different sheddases can also influence ADAM17 selectivity. Tetraspanins regulate the substrate selectivity of ADAM17 and iRhoms interactivity [47], with iRhom2 principally related to the inflammatory process [55, 56]. The regulation of ADAM17 activity is also achieved through its degradation. After fulfilling its functions, ADAM17 can be internalized from the cell surface through clathrin-dependent internalization and subsequent recycling or degradation [46]. This process helps maintain a balanced level of ADAM17 activity in the cell and prevents excessive proteolysis. A regulator that determines the fate of ADAM17 after internalization in resting cells was recently described. Phosphofurin Acidic Cluster Sorting Protein 2 (PACS-2) diverts ADAM17 away from degradation and instead promotes the recycling of the protease [57]. Also, iRhoms stabilize the ADAM17 membrane complex [58, 59].

One challenge of using chemical inhibitors to target ADAM17 is the similarity of its catalytic domain to other proteases in the metzincin superfamily. This similarity can lead to off-target effects and a lack of specificity when using inhibitors, making it challenging to develop effective and selective drugs for ADAM17. Consequently, after examining the primary regulatory processes of ADAM17, iRhoms has emerged as a crucial factor in managing ADAM17's activity through three main mechanisms: maturation, substrate selectivity, and stabilization. Given the distinct cellular expression and substrate selectivity of iRhom2, primarily found in macrophages [50, 60] and associated with TNF-α release associated with ADAM17 [55], iRhom2 emerges as a potentially viable target for modulating ADAM17 function. This approach could surmount the challenge related to the off-target effects of ADAM17 targeting.

### 2.3 Function

ADAM17 was the first sheddase to be characterized. This enzyme mediates the ectodomain shedding of over 80 substrates, including cytokines, growth factors, adhesion molecules, and endocytic receptors [44]. Due to its numerous substrates, ADAM17 is involved in several biological processes, such as development, regeneration, immunity, chronic inflammation, and tumorigenesis [61-63]. In this review, we will focus on the physiological and pathological functions of ADAM17 that have been characterized *in vivo*, particularly in the context of metabolic and neurodegenerative diseases.

As a general mechanism, ADAM17 generates two potent initiators of the immune response: the soluble IL-6 receptor (IL-6R) and TNF-α. Consequently, it represents a key component in the pathophysiology of autoimmune and chronic diseases [64, 65]. In neutrophils and macrophages, ADAM17 controls the cleavage of membrane-bound TNF-α into pro-inflammatory soluble TNF-α (sTNF-α) and cleavage of TNF-Receptor (TNF-R) into sTNFR. This process is tightly regulated by iRhom1 and iRhom2 and Polo-like kinases [66, 67], which have already been described previously.

Due to its involvement in various physiological and pathological processes, ADAM17 knock-out mice often die within several hours after birth, indicating that the loss of ADAM17 is not compatible with life [61]. The first conditional ADAM17 knock-out mice were reported by Blobel and coworkers in 2005 [68]. They inactivated the ADAM17 gene in myeloid cells and demonstrated that the loss of ADAM17 prevented death from lethal endotoxin injection. Furthermore, numerous groups have used the conditional ADAM17 knock-out mice to inactivate the ADAM17 gene in various tissues, demonstrating the essential role of ADAM17 in the skin, heart, liver, and innate and acquired immunity.

## 3 ROLE IN DIABETES

The involvement of ADAM17 in the development and progression of diabetes is well established. ADAM17 substrates are directly involved in the progression of T2DM, primarily through the dysregulation of inflammation. The pro-inflammatory cytokine TNF-α is linked to obesity, inflammation, and insulin resistance due to its crucial contribution to adipocyte metabolic dysregulation [47, 69]. Elevated TNF-α results in the serine phosphorylation of insulin-receptor substrate-1 (IRS-1), which facilitates the ubiquitination of this important effector downstream of the insulin receptor kinase, consequently blunting insulin signaling [32]. Shedding of the IL-6 receptor (IL-6R) is related to the IL-6 trans-signaling pathway, which is also linked to obesity-induced adipose tissue inflammation [70]. IL-6 causes insulin resistance by impairing the phosphorylation of insulin receptors and IRS-1 *via* the overexpression of SOCS-3 (Suppressor of cytokine singling 3) [71].

Additionally, ADAM17 indirectly enhances IL-1 signaling in cells by selectively cleaving the decoy receptor IL-1R2, which promotes IL-1 binding to IL-1R1 [72]. By altering the balance between IL-1R1 and its decoy receptor IL-1R2, ADAM17 enhances sensitivity to IL-1, leading to the activation of nuclear factor-kappa B (NF-κB) and promoting a major pro-inflammatory pathway, contributing to the pathogenesis of insulin resistance. Finally, ADAM17 cleaves pre-adipocyte factor 1, which inhibits adipose tissue differentiation, reduces the expression of adipocyte markers, and decreases fat mass [73].

In humans, ADAM17 expression and enzymatic activity were increased in T2DM skeletal muscle, as were the substrates TNF-α and IL6-R, which positively correlated with insulin resistance [74]. In experimental studies, treatment with the ADAM17 inhibitor Marimastat improved surrogate markers for insulin sensitivity and reversed steatosis in mouse models of diet-induced obesity and leptin deficiency [75]. Inactivation of ADAM17 suppressed high-fat diet (HFD) induced obesity, insulin resistance, hepatosteatosis, and adipose tissue remodeling in mice, with increased energy expenditure, suggesting an essential role for ADAM17 in the development of obesity-induced metabolic disorders [76]. Furthermore, systemic overexpression of ADAM17 induced macrophage infiltration and subsequent fibrosis in adipose tissue under a high-fat diet regimen, increased TNF-α serum levels, general inflammation, and macrophage-related cytokines (INF-y, IL-1b, MCP-1) [77], demonstrating the sufficient actions of this protease in the development of T2DM.

Regarding tissue influence, visceral adipose tissue (VAT) was the only tissue to increase ADAM17 activity in response to the development of obesity [78]. However, the loss of adipocyte ADAM17 played no evident role in baseline metabolic response when mice were challenged with HFD [79]. The ADAM17 silencing of VAT macrophage-targeted was sufficient to reduce and alleviate visceral inflammation and improve T2DM by reducing whole-body inflammation and improving insulin resistance in an obesity-induced diabetes model [80]. Table **1** provides the highlights of previously published studies related to the involvement of ADAM17 in diabetes development [81-84].

## 4 INVOLVEMENT IN NEURODEGENERATIVE DISEASES

ADAM17's involvement in the progression of brain disease is considered a double-edged sword due to its two distinct functions: (1) the regulation of amyloid precursor protein (APP), which is fundamental to preventing the amyloid formation in AD, and (2) the promotion of neuroinflammation, which is also linked to critical mechanisms driving AD progression. Given its crucial role in orchestrating APP shedding and TNF-α responses, it is reasonable to speculate that ADAM17 may exert dual and opposing effects on the development of neurodegenerative diseases. Neuron-associated ADAM17 could have a beneficial impact by triggering the non-amyloidogenic pathway of APP processing. At the same time, microglia-associated ADAM17 might be detrimental due to its ability to release TNF-α and sustain chronic inflammatory responses.

In the context of AD, the prevailing hypothesis places amyloid-beta (Aβ) accumulation at the center of the disease's pathogenesis. Aβ originates from APP through sequential proteolytic cleavage. APP is a type I transmembrane protein that can be processed through two distinct pathways: the amyloid and non-amyloid pathways. In the amyloid pathway, proteolytic processing by β- and γ-secretases generates neurotoxic Aβ from APP [85]. Conversely, in the non-amyloid pathway, ADAM17 exhibits α-secretase activity that cleaves APP within the Aβ domain, resulting in the release of the soluble APP alpha fragment (sAPPα) and consequently preventing the production of neurotoxic Aβ [60, 86]. Notably, a preclinical study using abemaciclib mesylate to treat an Aβ-overexpressing mouse model of AD demonstrated improved spatial and recognition memory in treated animals and decreased Aβ accumulation. This effect was attributed to the enhanced activity of ADAM17 [87]. Additionally, reduced ADAM17 function has been linked to Aβ accumulation, short-term memory, and cognitive deficits in mice [88, 89].

Furthermore, ADAM17's role extends to modulating the shedding of the triggering receptor expressed on myeloid cell 2 (TREM2) [90]. TREM2 facilitates microglial phagocytosis, which is crucial for managing amyloid plaques [91]. The shedding of TREM2 by ADAM17 impairs this function, leading to dysregulation of amyloid phagocytosis and accumulation of Aβ. Interestingly, ADAM17 expression levels are elevated in AD patients compared to healthy individuals, with a significant correlation between elevated plasma ADAM17 activity and cognitive decline in AD patients [92, 93].

Beyond APP processing, ADAM17 also plays an active role in neuroinflammation and AD-related microglial activation [94]. ADAM17 is constitutively expressed in microglia and may promote microglial cell survival [95]. Furthermore, it is involved in the generation and maturation of several AD-related inflammatory factors, such as TNF-α, EGF-like growth factors, and specific cell adhesion molecules (CAMs) [68]. Imaging studies have shown that reactive microglia can be detected at very early clinical stages of the disease [96]. Also, microglial activation was observed in AD mouse models before amyloid plaque formation [97]. The role of inflammation in AD pathogenesis is further supported by studies demonstrating the efficacy of TNF inhibitors in reducing plaque deposition and microglial activation in both preclinical and clinical AD models [98].

ADAM17 modulates the expression of cell adhesion molecules, including VCAM-1 and ICAM-1 [99, 100], which are involved in leukocyte migration across the BBB and infiltration into the CNS [101]. Additionally, ADAM17 cleavage of CX3CL1 (Fraktaline) [102], another adhesion molecule with both neuroprotective and neurodegenerative roles, highlights its complex involvement in central nervous system (CNS) processes.

Animal model studies have further elucidated ADAM17's role in neurodegenerative diseases, showcasing its intricate interplay within the CNS. Transgenic and knockout models specifically designed to overexpress or ablate ADAM17 in CNS cells and brain tissue have provided critical insights into its physiological and pathological implications. A study exploring the impact of ADAM17 knockout in astrocytes showed an amelioration of HIV-1 Tat-induced inflammatory responses and neuronal death, suggesting the enzyme's involvement in neuroinflammatory pathways relevant to neurodegenerative diseases [103]. Furthermore, research on a loss-of-function variant of ADAM17 associated with familial Alzheimer's disease highlighted the enzyme's genetic implications in neurodegeneration, offering a genetic perspective on its role in these diseases [104]. On the other hand, a study in the APP/PS1 mouse model of Alzheimer's disease demonstrated that overexpression of ADAM17 could influence cerebrovascular functions and cognitive abilities, highlighting its potential role in AD pathology and as a therapeutic target [105]. Table **2** outlines the key studies related to the involvement of ADAM17 in AD pathology [106].

**Fig. (1):**
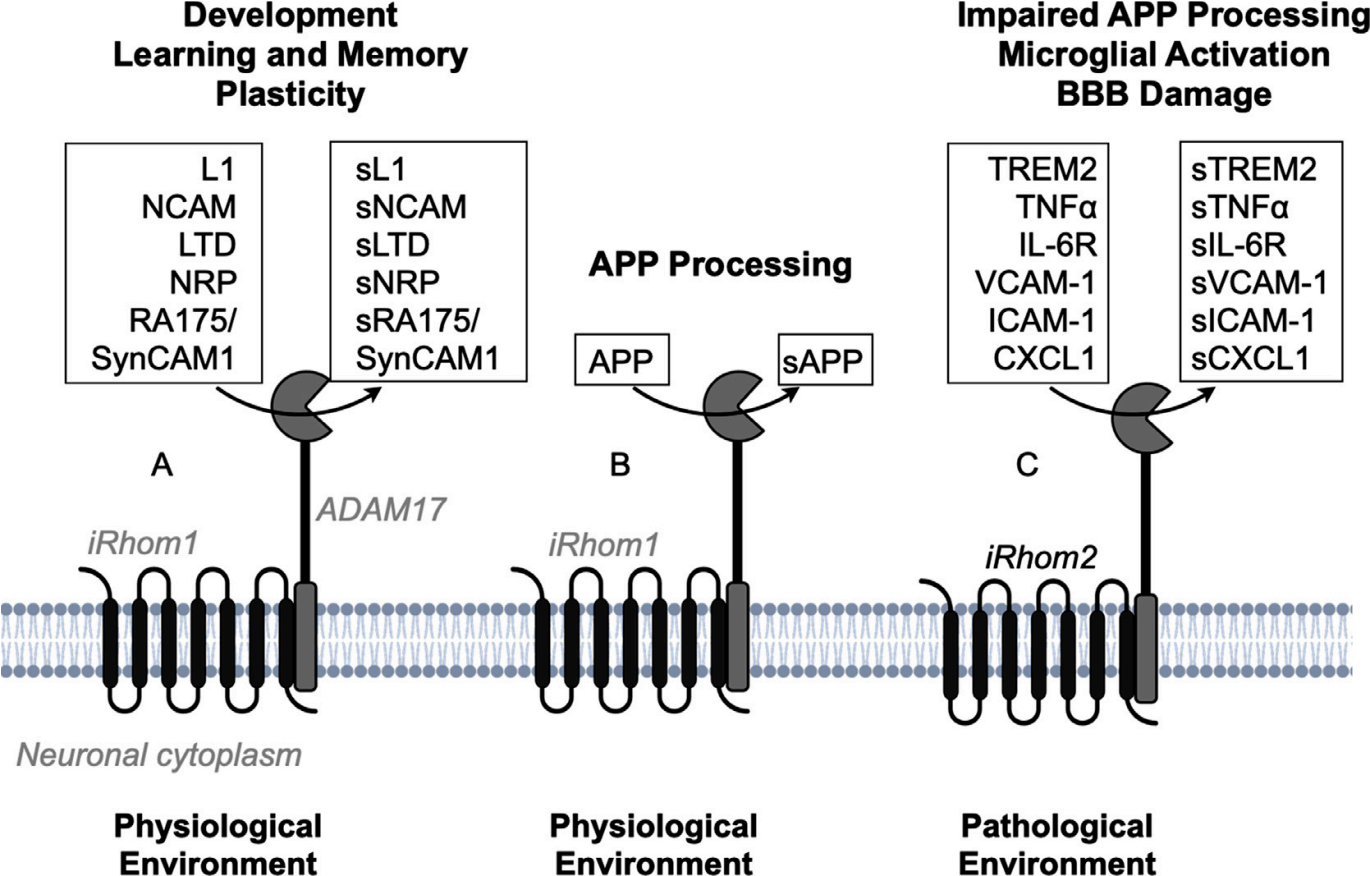
Involvement of ADAM17 in brain physiology (**A**, **B**) and neurodegenerative disease (**C**). (**A**) ADAM17 cleaves a series of proteins related to neural development (L1 and NCAM), learning and memory (LTD and NRP), and plasticity (RA175/SynCAM1) is regulated by iRhom1. (**B**) In neurons, iRhom1 may have a beneficial function as ADAM17 is responsible for processing APP into a non-amyloid form, known as sAAPα. (**C**) In microglia cells, protein processing can lead to impairment of APP processing (consequent to cleavage by TREM2), microglia activation (due to the release of pro-inflammatory cytokines, such as TNF-α and IL-6R and leukocyte), and upregulated inflammatory response (due to the cleave of adhesion molecules such as VCAM-1, ICAM-1 and CXCL1) causing damage to the blood-brain barrier damage.

Further, ADAM17's regulatory mechanisms involve its interaction with iRhom1 and iRhom2, which differ in expression across cell types. Specifically, microglia predominantly express iRhom2, which is involved in inflammatory actions, while iRhom1 is ubiquitously expressed throughout most brain cells [50, 60]. Given this context, iRhoms represents a promising therapeutic target in neurodegenerative diseases. Due to their distinct tissue expression, ADAM17's ability to process APP or TNF-α can be differentially regulated by either iRhom1 or iRhom2 [107]. In line with its role in promoting TNF-α release and neuroinflammation, iRhom2 has been identified as a genetic risk factor in AD [108]. Consequently, a potential inhibition of iRhom2 would inactivate ADAM17 in microglia, thereby preventing the pathological cleavage of TNF-α. However, in neurons, iRhom1 would still support the ADAM17-dependent non-amyloidogenic processing of APP and maintain the other physiological functions of the protease in the brain (Fig. **1**).

## 5 IMPACT OF DIABETES ON COGNITIVE IMPAIRMENT

The interplay between metabolic dysregulation, inflammation, and oxidative stress in T2DM contributes to cognitive decline and an increased risk of neurodegenerative diseases. Glial cells, which include astrocytes, microglia, and oligodendrocytes, are crucial for maintaining brain homeostasis and supporting neuronal functions. In T2DM, these cells experience adverse effects due to significant changes in the brain environment, resulting in impaired neuronal support and exacerbated neuroinflammation [109].

Chronic low-grade inflammation in T2DM leads to the activation of microglia [110], the resident immune cells of the central nervous system. This activation releases pro-inflammatory cytokines, such as TNF-α, IL-6, and IL-1β, which worsen neuroinflammation, contribute to neuronal damage, and promote cognitive decline [110]. Moreover, T2DM affects astrocytes, which are responsible for maintaining the blood-brain barrier and providing metabolic support to neurons. Insulin resistance and subsequent chronic hyperglycemia can alter astrocyte morphology and function, compromising neurovascular coupling, reducing neurotrophic support, and disrupting glutamate homeostasis [111]. Additionally, hyperglycemia-induced oxidative stress and inflammation impair the function of oligodendrocytes, which are responsible for myelin production and maintenance. This impairment leads to demyelination, reduced neuronal signal transmission, and neurodegeneration [112]. Also, insulin plays a crucial role in the brain's management of Aβ plaques. The insulin-degrading enzyme, responsible for breaking down insulin and Aβ, may prioritize insulin over Aβ when insulin levels are high, leading to Aβ accumulation [113]. Moreover, insulin maintains the blood-brain barrier and consequently enhances cerebral perfusion, which is essential for Aβ clearance [114].

In summary, ADAM17 is involved in the shedding of membrane-bound proteins, including pro-inflammatory cytokines and their receptors. This process is crucial in modulating inflammatory responses and insulin signaling pathways, both of which are key contributors to the development of cognitive deficits in T2DM patients. The activation of ADAM17 in diabetes can lead to an exacerbation of inflammatory and oxidative stress responses, thereby influencing glial cell function and neuronal integrity, which are essential in the context of cognitive health.

## 6 ADAM17 RELATED SIGNALING PATHWAYS

**Fig. (2):**
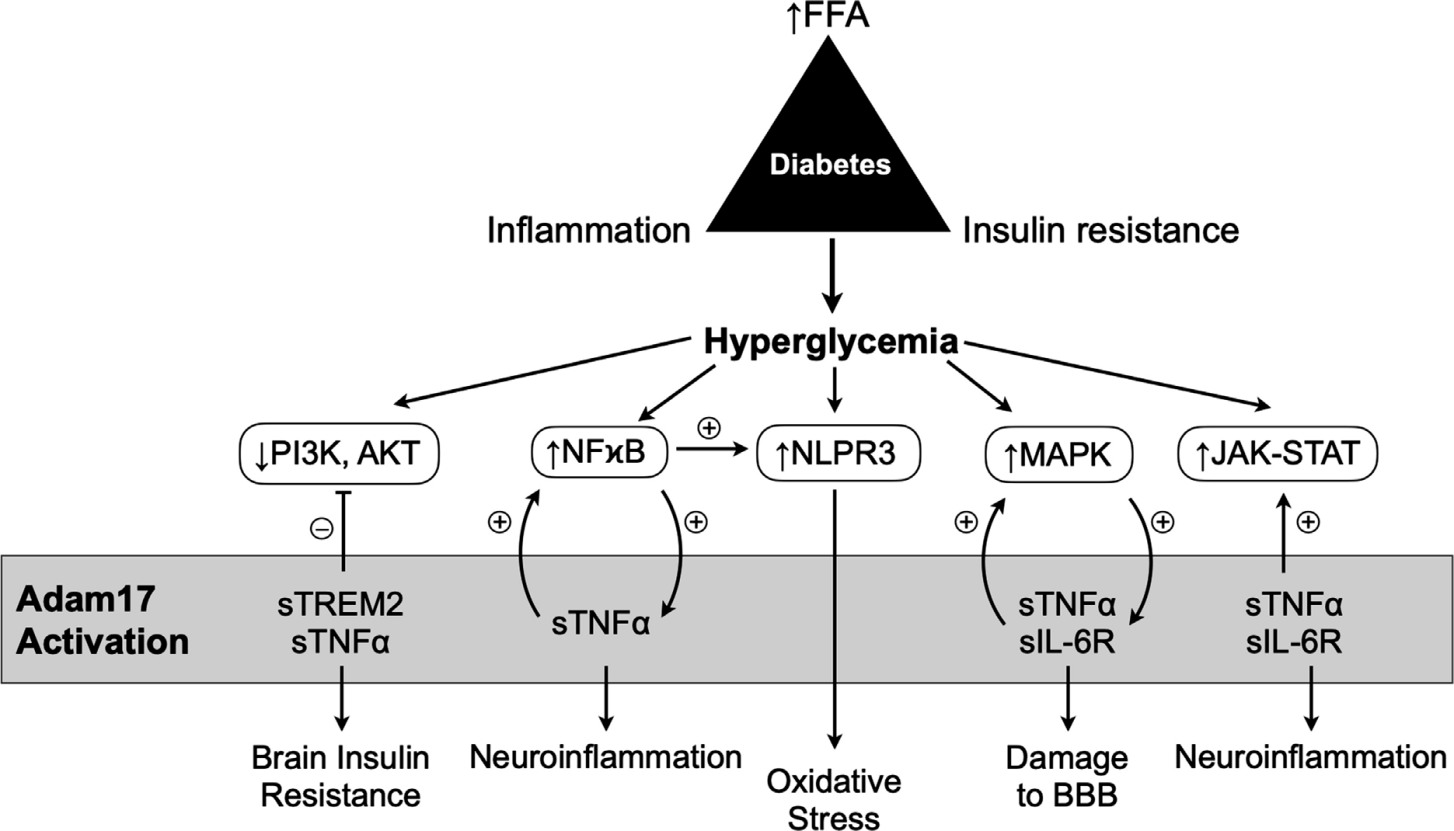
Crosstalk mechanisms related to diabetes, cognitive impairment, and ADAM17. The trilogy of diabetes consisted of increased concentration of free-fat-acids (FFA), development of chronic inflammation, and consequent insulin resistance, which leads to hyperglycemia. Hyperglycemia causes dysregulation of cell signaling pathways related to insulin resistance (PI3K/AKT), inflammation (NF-kB, MAPK, and JAK-STAT), and oxidative stress (NLPR3), which eventually will lead to the activation of glial cells and subsequent neurodegeneration. The primary substrates cleaved by ADAM17 include cytokines TNF-α and IL-6R, which cause increased activation of NF-kB, NLPR3, MAPK, and JAK-STAT and damage the blood-brain barrier and neuroinflammation. The increased shedding of TNF-α, and TREM2 by ADAM17 will accentuate the inhibition of the PI3K/AKT pathway, leading to greater impairment in brain insulin resistance.

ADAM17 plays a critical role in the modulation of signaling pathways that are pivotal in the pathophysiology of diabetes and its associated-neurodegenerative consequences. ADAM17 affects several signaling pathways involved in stress response, including the phosphatidylinositol-3-kinase and protein kinase B (PI3K/AKT), NF-κB, Janus kinase-Signal Transducer and Activator of Transcription (JAK-STAT), mitogen-activated protein kinase (MAPK), NOD-like receptor family, and pyrin domain containing 3 (NLRP3) inflammasome signaling pathways (Fig. **2**).

## 7 PI3K/AKT PATHWAY

The PI3K/AKT signaling pathway is instrumental in promoting anti-inflammatory, anti-oxidative, and anti-apoptotic responses in neurons [115]. In the milieu of T2DM, elevated chronic plasma levels of TNF-α, a consequence of ADAM17's shedding activity, promote insulin resistance [116, 117], thereby decreasing the activation of the PI3K/AKT pathway [20]. Although the brain's insulin signaling is primarily considered independent due to the predominant presence of GLUT-1 and GLUT-3 over insulin-sensitive GLUT-4 [118], recent findings have highlighted a strong linkage between insulin resistance and cognitive impairments in diseases like MCI [119] and Parkinson’s Disease (PD) [120], with abnormalities in insulin receptor expression and AKT signaling. ADAM17 exacerbates this issue by not only increasing the proinflammatory cytokine profile but also by cleaving TREM2, further disrupting PI3K/AKT signaling and amplifying neuronal damage [121].

## 8 NF-κB PATHWAY

The NF-κB pathway, when activated by hyperglycemia-induced insulin resistance, leads to the production of pro-inflammatory cytokines and mediators in microglia alongside an increase in reactive oxygen species (ROS), impairing mitochondrial function and inducing neuronal damage [122]. Astrocyte polarization, connected to the NF-κB signaling pathway, further contributes to ROS production and pathological damage through reactive astrocytes activating the NF-κB downstream pathway [123-127] ADAM17's role in this context is to cleave membrane-bound TNF-α, releasing its soluble form that activates the NF-κB pathway [128], thus creating a feedback loop that exacerbates the inflammatory response and tissue damage [103, 129].

## 9 NLRP3 PATHWAY

NLRP3 inflammasome pathway is involved in diabetes development due to its influence on glucose tolerance, insulin resistance, inflammation, and apoptosis mediated in adipose tissue. Also, in the brain, a hyperglycemic environment activates pyroptosis, an inflammatory type of cell death, by increasing the expression of NLRP3 [130, 131]. In age-related neurological diseases, such as PD and AD, dopaminergic neurons can exhibit increased pro-inflammatory NLRP3 inflammasome activity [132]. In experiments using activating mutations, mice with heightened NLRP3 expression showed accelerated progression of motor deficits [132].

ADAM17 has been linked to the activation of the NLRP3 through a priming mechanism since ADAM17 mediated TNF-α shedding can activate the NF-κB pathway, which in turn upregulates NLRP3 expression and primes the inflammasome for activation [133]. Interestingly, Madhu *et al.* (2021) observed that melatonin supplementation was efficacious for improving cognitive and mood function in rats committed to chronic Gulf War illness through the reduction of oxidative stress and NLRP3 inflammasome pathway. This promising result can further be linked to the research conducted by Zhang *et al.* 2022, in which results demonstrate a beneficial effect of melatonin in hippocampal inflammation was associated with inhibiting ADAM17/TNFα axis [106].

## 10 MAPK PATHWAY

The MAPK signaling pathway is implicated in the pathogenesis of diabetes and its complications through hyperglycemia and metabolic factors that activate ERK, JNK, and p38 MAPK [134]. p38 MAPK activation has been implicated in the development of diabetic complications, such as nephropathy and retinopathy, through the promotion of inflammation and endothelial dysfunction due to its significant role in the recruitment of leukocytes to sites of inflammation [135]. In neurodegeneration, the MAPK pathway is tied to microglial activation and inflammatory mediator production [136, 137]. ADAM17 influences this pathway by modulating its activation through the phosphorylation of its cytoplasmic domain, affecting the balance between ADAM17 dimers and monomers [138].

In the absence of MAPK stimulation, ADAM17 exists as dimers at the cell surface, enabling TIMP3 to interact efficiently with and inhibit ADAM17. However, the activation of MAPK signaling leads to increased monomer presentation and the release of TIMP3 from ADAM17 [138], which results in the enhanced production of pro-inflammatory signaling and a positive feedback loop between the MAPK and ADAM17 pathways. Also, the deleterious impact of TNF-α on insulin signaling occurs in a p38 MAPK-dependent manner [32, 139]. This interplay between ADAM17 and MAPK signaling underscores the complex nature of their regulatory mechanisms and highlights the potential for therapeutic interventions targeting these interconnected pathways.

## 11 JAK-STAT PATHWAY

The JAK-STAT pathway is a crucial cell signaling pathway involved in the regulation of various cytokines and growth factors, including TNF-α, which plays a central role in diabetes development and neuroinflammation, contributing to the development of MCI [140]. In the JAK-STAT pathway, TNF-α binding to its receptor on the surface of cells activates JAKs, which, in turn, activate STAT proteins and lead to the expression of pro-inflammatory genes. STAT3 phosphorylation and activation by JAKs have been demonstrated in a variety of neurodegenerative disease models and shown to play a role in damage repair, cell survival, and scar formation [141].

ADAM17 is involved in the shedding of cytokine receptors, such as IL-6R, leading to the formation of sIL-6R, which can stimulate the JAK/STAT signaling pathway through a process called trans-signaling [142]. In diabetes, increased ADAM17 activity and the subsequent activation of JAK/STAT signaling *via* sIL-6R trans-signaling have been associated with insulin resistance, inflammation, and the development of diabetic complications [143]. Similarly, in neurodegenerative diseases such as AD and PD, activating the JAK/STAT pathway by ADAM17-mediated shedding of cytokine receptors contributes to neuroinflammation and neuronal dysfunction [144]. Therefore, understanding the relationship between ADAM17 function and the JAK/STAT pathway in diabetes and neurodegenerative diseases can provide insights into potential therapeutic strategies targeting this interplay to alleviate disease symptoms and progression.

## 12 ADAM17 AS A PROSPECTIVE THERAPEUTIC TARGET

Studies investigating the effects of anti-diabetes drugs on cognitive function suggest that these drugs could improve cognitive function to varying degrees despite some controversial findings [145]. However, it is still debatable whether anti-diabetes drugs can alleviate or even prevent diabetes-associated MCI. The primary clinically used anti-diabetes drugs are sulfonylureas, biguanides, α-glucosidase inhibitors (AGIs), thiazolidinediones (TZDs), sodium-glucose cotransporter type 2 inhibitors (SGLT2i), dipeptidyl peptidase-4 inhibitors (DPP-4Is), glucagon-like peptide-1 receptor agonists (GLP-1RAs), and insulin analogs [146].

Sulfonylureas stimulate insulin secretion and have shown potential in reducing neurotoxicity and improving learning and memory in rodent models [147, 148]. However, their impact on cognitive function in clinical settings remains unclear, with some studies showing reduced dementia risk [123] and others showing increased PD risk in T2DM patients [149]. Furthermore, the risk of hypoglycemia associated with sulfonylureas can have detrimental effects on cognitive functions.

Metformin, a biguanide drug, is the first-line treatment for T2DM. Metformin offers various beneficial effects, including anti-diabetic, anti-cancer, neuroprotective, and life span extension properties [150]. Although some studies report that patients with T2DM taking metformin exhibited worse cognitive performance than those not taking the drug [120, 151, 152]. Its use has been shown to improve cognitive function in T2DM models [153] and, in clinical studies, to slow down the progression or even prevent diabetes-associated MCI [154-156] in different epidemiologic and meta-analysis studies through the years [157, 158].

Thiazolidinediones (TZDs) such as pioglitazone and rosiglitazone are considered a class of anti-hyperglycemic agents and agonists of peroxisome proliferator-activated receptor-gamma (PPARγ); they have potential neuroprotective effects due to their anti-inflammatory and anti-oxidation properties [159]. However, initial studies demonstrate that in cognitive impairment [160, 161] the mechanism of action in MCI can be elucidated. The increased risk of cardiovascular adverse effects may preclude the extended use of thiazolidinediones.

GLP1-RAs and DDP-4Is are newer oral antidiabetic drugs prescribed to people with T2DM and have demonstrated neuroprotective effects in various studies. GLP1-RAs stimulate the pancreas to release insulin, while DDP-4Is slow the inactivation and degradation of GLP-1. Both drug classes target GLP-1 and have shown benefits in neurodegenerative diseases such as AD, PD, and T2DM-associated cognitive decline [162-165]. The neuroprotective effects of GLP-1RAs are attributed to multiple mechanisms, including stimulating neurotrophic factors, restoring cerebral insulin signaling, and suppressing inflammation and oxidative stress [166]. DPP-4Is, such as sitagliptin, have demonstrated neuroprotective effects in AD, PD, and HD experimental models [167-170]. They have also shown potential for improving cognitive function in neurodegenerative diseases.

Insulin plays a crucial role in cognition, and some studies have shown that insulin administration improves memory in AD patients [171]. However, long-term intensive insulin treatment has potential side effects [172], and more research is needed to determine its safety and efficacy in cognitive improvement.

SGLT2i are anti-diabetes agents with potential neuroprotective effects, as shown in preclinical studies [173]. A recent study found that SGLT2i empagliflozin improved cognitive and physical impairment in older adults with T2DM and heart failure [174], sparking interest in further investigation into the potential neuroprotective effects of SGLT2i. Anti-diabetes drugs have shown neuroprotective effects in T2DM patients with or without neurodegenerative diseases, suggesting their potential repurposing for treating such conditions. However, some studies found that these drugs did not improve or even worsen neurodegenerative disease progression [152, 175].

In this context, a comprehensive understanding of the most effective strategies for preserving cognitive function in diabetic patients, particularly in relation to ADAM17's involvement in diabetes-associated MCI, necessitates continued investigation into these treatments and the development of targeted therapies. Consequently, further research is essential to pinpoint the most effective strategies for maintaining cognitive function in this patient population.

ADAM17 pathway inhibition is a promising therapeutic approach for neuroinflammatory conditions. One of the significant benefits of this approach is its ability to improve control over inflammation signaling pathways without affecting the anti-inflammatory TNFR2 pathway [62]. Due to the general involvement of ADAM17 in the principal signaling pathways involving brain damage associated with diabetic MCI, it is postulated that selectively inhibiting the ADAM17 pathway would have significant implications for the modulation of neuroinflammation.

Developing ADAM17 inhibitors for clinical use presents several challenges, primarily due to the complexity of ADAM17's functions, its involvement in various signaling pathways, and structural similarities with other ADAM family proteins. In this way, inhibitors with poor specificity may cause off-target effects, leading to unintended consequences and potential side effects. Addressing these challenges is crucial for successfully developing ADAM17 inhibitors for clinical use. Also, ADAM17's functions in AD are complex and somewhat contradictory. While ADAM17 is involved in the non-amyloidogenic processing of APP, which is considered a neuroprotective pathway, it also promotes neuroinflammation [60], which exacerbates neuronal damage and synaptic dysfunction.

In this way, the presence of iRhom2 in a brain-specific distribution within microglia [49, 50, 59] is an exciting development in the neuroinflammation research. This distribution offers greater specificity and potentially fewer adverse effects than previously reported methods (Fig. **1**). As presented in this article, microglia cells are the primary immune cells of the central nervous system and play a critical role in neuroinflammation. By targeting iRhom2 within these cells, the ADAM17 pathway can be more effectively inhibited to attenuate inflammation without interfering with other essential functions of microglia cells and pathways related to APP processing by ADAM17.

Although promising evidence supports inhibiting the iRhom2/ADAM17 pathway, further research is necessary to establish its safety and efficacy. Proposed experimental approaches could involve *in vitro* studies to investigate the effects of ADAM17 pathway inhibition on neuroinflammation and potential adverse effects on microglia cells. Additionally, pre-clinical models could be utilized to evaluate the efficacy and safety of inhibitors of the iRhom2 pathway. Investigating the involvement of the ADAM17/iRhom2 pathway in the development of cognitive impairment related to neuroinflammation has significant potential for the field of neuroscience, as it may offer insights into the underlying mechanisms of neurodegenerative diseases such as AD, PD, and multiple sclerosis.

## CONCLUSION

ADAM17 is a transmembrane protein that plays a significant role in various biological processes, including inflammation, cell proliferation, and tissue regeneration. It acts as a sheddase, releasing bioactive molecules, such as cytokines, growth factors, and receptors, by cleaving the extracellular domain of transmembrane proteins. This process has been linked to the development of several disorders, making ADAM17 a crucial target for therapeutic interventions.

ADAM17 plays a direct role in the pathogenesis of diabetes-associated neurodegenerative processes, including the cell signaling pathways involving both diseases, such as AKT, NF-κB, JAK-STAT, MAPK, and NLRP3 inflammasome pathways. Thus, targeting ADAM17 represents a promising approach for treating cognitive impairment and neurodegenerative diseases. Moreover, identifying new targets within this pathway could lead to developing novel therapeutic strategies that specifically target inflammation without interfering with other essential immune system functions. One promising regulator protein that has shown potential in modulating ADAM17 activity in metabolic diseases is iRhom2.

Targeting iRhom2 could be a promising therapeutic approach for MCI, given that most current treatment options are related to metabolic impairment caused by diabetes. By targeting ADAM17 through iRhom2 modulation, the ADAM17 pathway can more effectively inhibit and reduce inflammation without interfering with other essential functions of microglia cells and pathways related to APP processing by ADAM17, being a viable future target for MCI.

## Figures and Tables

**Table 1:** Studies illustrating the role of ADAM17 in the pathophysiology of diabetes.

Study	Species	Condition	Intervention	Assessment	Main Findings
Maekawa *et al*. (2019) [81]	Mice	T1DM and T2DM	Intraperitoneal injection of an ADAM17 inhibitor (JTP 96193) once daily for seven days	Enzymatic activity Kit of ADAM17	Inhibition of ADAM17 prevented development insulin resistance in T2DM and peripheral neuropathy in T1DM
Yong *et al*. (2017) [80]	Mice	T2DM associated with obesity	Visceral adipose tissue macrophage targeted ADAM17 silencing	Indirect access of ADAM17 function was accessed through quantification of inflammatory cytokines	ADAM17 gene silencing in visceral macrophages alleviated visceral fat inflammation and improved T2DM
Kawasaki *et al*. (2013) [78]	Mice	Early stage of obesity	No intervention	Enzymatic activity Kit of ADAM17	In early stage of obesity ADAM17 activity is elevated only in visceral adipose tissue
De Meijer *et al*. (2011) [75]	Mice	Hepatic steatosis and Insulin resistance	Orally administration of an ADAM17 inhibitor (Marimastat) twice daily for two weeks	α-Secretase activity assay for ADAM17	ADAM17 inhibitor improved insulin sensitivity and reversed steatosis in mouse models of diet-induced obesity
Kaneko *et al*. (2011) [76]	Mice	T2DM associated with obesity	Transgenic mice with temporal systemic ADAM17 deletion	No direct assays were used to access ADAM17 involvement	Inactivation of ADAM17 suppressed diet-induced obesity, insulin resistance, hepatic steatosis, and adipose tissue remodeling
Togashi *et al*. (2002) [82]	Rat	Nonobese insulin-resistant hypertensives	Intraperitoneal injection of an ADAM17 inhibitor (KB-R7785) once daily for two weeks	No direct assays were used to access ADAM17 involvement	ADAM17 plays a major role in insulin resistance in nonobese insulin-resistant models
Prasad *et al*. (2022) [83]	Rat	Aorta inflammation associated with T1DM	Orally administration of diosgenin once daily for four weeks	mRNA and protein expression of iRhom2/ADAM17, *via* PCR and WB respectively	By regulating iRhom2/ADAM17 signaling, diosgenin lowered dyslipidemia, hypertension, and inflammation in aorta of T1DM rats.
Lownik *et al*. (2020) [79]	Mice	Obesity	Adipocyte-specific ADAM17 knockout model	No direct assays were used to access ADAM17 involvement	Loss of adipocyte ADAM17 plays no evident role in baseline metabolic responses
Serino *et al*. (2007) [84]	Mice	T2DM associated with obesity	Heterozygous mice for ADAM17	No direct assays were used to access ADAM17 involvement	ADAM17 heterozygous mice presented protection against T2DM associated with obesity

**Table 2:** Studies illustrating the role of ADAM17 in AD and neuroinflammation.

**Study**	**Species**	**Condition**	**Intervention**	**ADAM17 Involvement**	**Main Finding**
Tian *et al*. (2023) [105]	Mice	AD	No intervention	Protein expression of ADAM17 trough WB and IHC	Reduced ADAM17 expression in cerebral micro vessels may contribute to the development of cognitive dysfunction in AD
Skovronsky *et al*. (2001) [93]	Human	Control and AD samples	No intervention	Protein expression of ADAM17 trough WB and IHC	In control samples ADAM17 expression was main located in neurons and in AD samples its expression was colocalized with Aβ plaques formation
Pietri *et al*. (2013) [89]	Mice	Prion and AD	PDKI inhibition trough chemical and genetic deletion	ADAM17 activity access through indirect assessment of sTNF-α and expression pattern through IHC	PDK1 inhibition attenuates AD–like pathology and prion disease through ADAM17 upregulation
Sun *et al*. (2014) [92]	Human	AD	No intervention	ADAM17 expression and activity was accessed through WB and enzymatic activity kit respectively	ADAM17 activity is increased in patients with MCI and AD
Zhang *et al*. (2022) [106]	Rat	Chronic stress-induced hippocampal inflammation	Intraperitoneal injection of melatonin once daily for seven days	ADAM17 expression was accessed through WB	Melatonin relieves chronic stress-induced hippocampal inflammation by inhibiting ADAM17/TNF-α axis

## LIST OF ABBREVIATIONS

APP: Amyloid Precursor Protein
Aβ: Amyloid-beta
CAMs: Cell Adhesion Molecules
CNS: Central Nervous System
HFD: High-fat diet
IFNγ: Interferon-gamma
IL-1β: Interleukin-1 beta
IL-6: Interleukin-6
IRS-1: Insulin-receptor Substrate-1
MCI: Mild Cognitive Impairment
MMPs: Matrix Metalloproteases
MPD: Membrane-proximal Domain
NF-κB: Nuclear Factor-kappa B
PD: Parkinson’s Disease
ROS: Reactive Oxygen Species
T2DM: Type 2 Diabetes Mellitus
TNF-α: Tumor Necrosis Factor-alpha
TZDs: Thiazolidinediones
